# Interpersonal goals and social support network: examining the relation between perceived network density and burnout among nurses

**DOI:** 10.1186/s40359-025-03470-w

**Published:** 2025-10-16

**Authors:** Zena Toh, David S. Lee

**Affiliations:** 1 Department of Communication Studies, Christopher Newport University, 1 Avenue of the Arts, Newport News, VA 23606 USA; 2 Department of Communication, University at Buffalo, Buffalo, NY USA

**Keywords:** Interpersonal goals, Reducing burnout, Social support

## Abstract

**Background:**

Burnout remains a critical concern among nurses, yet relatively little is known about the motivational and relational mechanisms that may buffer against it. This study examined whether interpersonal goals influence how nurses perceive the density of their support networks and whether these perceptions are associated with burnout.

**Methods:**

A cross-sectional online survey was conducted with 220 registered nurses recruited through social media platforms. Burnout was assessed with the Professional Quality of Life scale, interpersonal goals were measured with established scales of compassionate and self-image goals, and perceived network density was measured with an ego-centered cognitive social structure generator. Regression-based mediation analyses using Hayes’ PROCESS macro were conducted to test hypotheses.

**Results:**

Nurses with higher compassionate goals perceived denser support networks, which were in turn correlated with lower burnout. Conversely, self-image goals correlated with sparser networks and higher burnout. Mediation analyses confirmed that network density accounted for the indirect association between interpersonal goals on burnout.

**Conclusions:**

Interpersonal goals were associated with how nurses cognitively represented their support networks, which was in turn associated with burnout. Future research should investigate whether fostering compassionate goals or strengthening perceptions of network interconnectedness is related to well-being.

Perceived social support, the belief that one can rely on others, is one of the strongest social factors that can buffer people from stress [1–3]. For example, perceived social support has been found to be related to enhanced psychological health during times of stress [4, 5], acting as a protective barrier against stressors [6]. More recently, Lee and colleagues [7] extended this work by showing that not only is perceiving one’s personal network members to be supportive beneficial, but viewing the network members to be supportive of one another has additional benefits for coping. A key question is then, what makes individuals view their personal network members to be supportive of one another? Answering this question seems important given emerging work showing that how people view and reflect on their network has important implications for social resources (see Bayer and colleagues’ work [8] for review). To this end, we incorporated research on interpersonal goals [9, 10] to understand how social motivation may be related to how people perceive their personal network.

## Interpersonal goals

Prior research on interpersonal goals [9, 11] has identified two distinct interpersonal goals that motivate how people think, feel, and behave in social contexts: Compassionate goals motivate people to promote the well-being of others; self-image goals drive people to focus on demonstrating their desirable images to others. Although research suggests that people with compassionate goals are more likely to perceive higher levels of support from their close relationships overall [9], no research has directly tested how they view the relationships among the members of their support network. Further, because most studies on interpersonal goals have examined perceptions of support in dyadic relationships, it remains unclear how these goals would be related with how people view their broader network.

To address those gaps in the literature, the current study examined how interpersonal goals are related with the perceived connectedness of people in one’s social circle, termed the density of one’s personal network. For example, if someone imagines that most of their friends know and interact with each other, they would have a closely interconnected network. On the other hand, if a person’s friends do not really know each other and rarely interact, they would have a less interconnected network. Building on the rich tradition of social support and coping research in the context of stress [2] (see Lakey and Cohen’s review on social-cognitive views of social support [12]), we further investigated whether perceived network density would be correlated with lower burnout among registered nurses whose recent work contexts have been related with heightened levels of stress [13, 14].

## Burnout within nurses

The healthcare profession has long been associated with high-stress environments, a challenge that was significantly exacerbated by the coronavirus disease-19 (herein referred to as COVID-19). The pandemic led to an increased workload for healthcare workers globally, especially nurses who are front-line workers during this critical period. Recent reviews found high levels of burnout symptoms amongst nurses globally [15, 16]. Defined as feelings of emotional exhaustion, cynicism, and ineffectiveness in relation to one’s work [17], burnout impacts nurses’ abilities to effectively care for their patients. It hinders their abilities to communicate and build rapport with their patients [18, 19], increasing chances of medical errors and compromising patient safety [20]. As such, identifying precise psychological mechanisms that can help reduce burnout of nurses is needed.

Nurses are particularly vulnerable to compassion fatigue (CF), which refers to the detrimental effects on people’s well-being in caring for others [21]. This concept was first introduced by Joinson [22] to describe limited ability to feel compassion due to chronic exhaustion from dealing with the suffering of others [23–26]. CF encompasses two distinct components: burnout and secondary traumatic stress (STS) [27]. Burnout is characterized by feelings of inadequacy and hopelessness that diminish one’s ability to perform job duties effectively, while STS involves emotional distress from exposure to others’ trauma [27, 28].

Recent studies have indicated high levels of burnout among nurses during the ongoing COVID-19 crisis [27, 29–32]. This is particularly concerning as burnout may cause nurses to become detached, apathetic, and depressed [33]. Studies have also shown that long-term negative outcomes include absenteeism, low morale, and high rates of nurse turnover [34, 35]. Consequently, it is important that burnout be recognized and addressed, with more insights into what can be done to treat it before it becomes problematic.

While most post-pandemic work (circa 2020) has examined how resilience is associated with reduced levels of burnout and STS [36–38], less is known about what relational factors may reduce or prevent feelings of burnout (see Labrague and De Los Santos’ work [39] for notable exception). For example, are there certain psychological mechanisms that could enhance one’s ability to leverage their close relationships and social resources to reduce levels of burnout? On the contrary, are there also psychological mechanisms that could limit one’s abilities to benefit from their social resources? To explore this, the current study looked at the relationship between interpersonal goals and people’s reflection on their social resources.

## Perception of social networks

A growing body of research suggests that people’s interpretations of their relationships has implications for the social resources they perceive to have (see Bayer and colleagues’ work [8] for review). At the heart of this research is the concept of cognitive social structure (CSS) [40], which refers to how individuals mentally represent and understand the patterns of relationships in their social network. CSS encompasses both how people perceive direct connections (e.g., their own relationships with others) and indirect connections (e.g., relationships between people they know).

Research has consistently shown that these mental representations of social networks have real consequences. For instance, several studies show that perceiving one’s network to be denser (i.e., seeing more connections among the people one knows) correlated with higher levels of perceived social support [7, 41, 42]. The way people mentally organize and understand their social networks can influence their ability to recognize and access available support resources.

While traditional social network research focused primarily on measuring the accuracy of people’s perceptions—how well they matched actual patterns of interaction [43]—CSS research takes a different approach. Instead of focusing on accuracy, CSS research examines how people’s mental representations of their social networks influence their thoughts, feelings, and behaviors [40]. For instance, a recent study demonstrated that simply thinking about one’s network as dense (versus sparse) led to higher levels of perceived social support, even after controlling for factors such as the quality of support received from individual network members or the types of relationships involved [7]. Importantly, this increased perception of social support enhanced people’s confidence in their ability to cope with stressors [7].

These findings suggest that beyond who people think of as part of their support network, how they mentally organize these relationships (i.e., seeing them as densely or sparsely connected) has meaningful implications for their perceived social resources and ability to cope with stress. This cognitive perspective on social networks provides a framework for understanding how people’s mental representations of their social world can shape their access to and utilization of social support. As such, in addition to whom people bring to mind, *how* people view the relationships among these individuals (i.e. dense vs. sparse) has implications for perceived social resources and coping under stress.

## Cognitive social structure and interpersonal goals

CSS research has argued that people vary in how they cognitively organize their relationships [43]. While existing research has examined the role of personality variables such as level of self-monitoring [44–46], preference for predictability [47], and individuals’ cognitive motivational traits (e.g. need for achievement [44]), less work has examined how social motivation is correlated with how people reflect on their personal network (see [44] and [48] for research on affiliative motivation and network cognition).

According to research on interpersonal goals [9, 10, 49], people with high levels of compassionate goals tend to approach relationships with a mutually beneficial mindset, whereby being supportive towards others is good for their relationships and for themselves. Additionally, people with compassionate goals often feel connected within their network as a result [10, 11]. However, no studies have directly tested whether compassionate goals are correlated with viewing the relationships among one’s personal network members as interconnected.

Additional theoretical perspectives corroborate the possibility that compassionate goals may be linked to denser perception of one’s network members. Studies show that concerns for others may influence perceptions of available social support [50, 51]. For instance, people who have greater compassion for others tend to perceive receiving equal amounts of compassion [50, 51]. Notably, reciprocity occurs between dyads whereby partners showed more compassion towards each other over time, correlating to increased feelings of connectedness, closeness, trust, and social support [9]. Given that people with compassionate goals tend to think of their relationships as mutually supportive of each other [11], it is possible that they would perceive a denser personal network when asked to reflect on their relationships with others. To that end, people with compassionate goals may be situated in a social system that allows them to better reap the benefits of their social resources. As such,

### H1

Compassionate goals will be positively correlated with perceived personal network density.

### H2

Compassionate goals will be negatively correlated with burnout.

### H3

Perceived personal network density will mediate the association between compassionate goals and burnout.

In contrast, people with high levels of self-image goals tend to focus on demonstrating their desirable images to others and approach relationships with a zero-sum mindset, whereby what is good for others can be considered a threat to themselves [9, 11]. Given this tendency, perceiving others to be supportive of one another may be perceived as threatening to people with high levels of self-image goals because less help would be available for them. Thus, they may be more motivated to see their personal network as less interconnected. As such,

### H4

Self-image goals will be negatively correlated with perceived personal network density.

Because the core motivation for self-image goals involves presenting one’s desirable qualities and hiding ones’ flaws, people with high self-image goals may experience feelings of disconnectedness and fatigue due to constant self-monitoring and impression management [9]. Further, prior studies have indicated that self-image goals are related with being less responsive to others [52–54]. Based on reciprocity norms, this may be related to reduced levels of social support received [9], which is crucial in mitigating burnout among nurses. Thus,

### H5

Self-image goals will be positively correlated with burnout.

Because higher levels of self-image goals may be negatively correlated with perceived density of the personal network, which may result in higher levels of reported burnout,

### H6

Perceived personal network sparsity will mediate the association between self-image goals and burnout.

## Overview of study

The current study examines how chronic interpersonal goals [52–54]—relatively stable individual differences in how people approach their relationships—are correlated with how nurses perceive their personal networks and the implications for social resources. Although interpersonal goals can fluctuate across situations, examining their enduring patterns is particularly relevant during sustained periods of stress. Building on prior research, we posited that chronic compassionate goals (vs. self-image goals) would be correlated with perceiving a denser personal network, thereby enabling individuals to access social resources that may help alleviate stress [2]. To provide a clear test of this framework, we investigated these hypotheses in the context of healthcare, where prolonged exposure to high-stress environments places nurses at elevated risk for burnout [15–17]. Specifically, we examined whether chronic interpersonal goals shape perceptions of one’s network as interconnected (vs. sparse), and whether these perceptions are correlated to lower levels of burnout. To this end, we conducted an online survey of professional nurses that assessed their interpersonal goals, personal network perceptions, and levels of burnout.

## Method

### Study design

The survey was conducted via Qualtrics and participants were recruited via social posts. Participants completed measures for ProQoL, which consisted of three subscales: Compassion Satisfaction (CS), burnout, and STS. Although the ProQoL measure consists of three subscales, the present study focused specifically on burnout as our construct of interest. They then answered questions pertaining to their personal network (as described below) and how much support they received from individuals in their support network. Importantly, we assessed burnout prior to measuring participants’ personal network; this was done intentionally to prevent potential survey order effects (e.g., burnout ratings influenced by certain relationships evoked in the personal network generator). Lastly, participants interpersonal goals were measured.

### Ethical consideration

All respondents indicated that they worked at a hospital at the time of taking the survey and provided their informed consent at the start of the study by clicking on the “I agree to participate” button. The authors’ Institutional Review Board at the University at Buffalo approved the study procedure prior to the start of the study (IRB ID: STUDY00005970).

### Participants

Prior to data collection, we conducted a power analysis based on the effect size (*f*^*2*^ = 0.15) from similar research [7]. This analysis indicated that a sample size of 112 participants would provide 80% power to detect a significant effect. We oversampled to ensure sufficient power and recruited a total of 248 registered nurses (*M*_age_= 33.13, *SD* = 12.04) via Instagram and Facebook. One hundred and forty respondents (56.5%) identified as females, 76 (30.6%) identified as males, while 32 (12.9%) preferred not to answer, with 1 identifying as nonbinary. A total of 113 respondents (45.5%) resided in Singapore, 130 (52.4%) from the United States, 2 (0.8%) from Malaysia, 2 (0.8%) from the United Kingdom, with one respondent declining to respond. For analytical purposes and since examining cross-cultural differences was not the focus of this study, we dummy coded country of residence into a dichotomous variable (0 = United States, 1 = Other countries). Respondents also indicated their nursing titles— a total of 218 (87.9%) identified as a Registered Nurse, 18 (7.2%) Licensed Practical Nurse, 8 (3.2%) Advanced Practice Registered Nurse, and 4 (1.6%) Nurse Managers.

### Outcome measures

#### Professional quality of life

Each of the three constructs (CS, burnout, STS) was measured on a 5-point scale (1 = *never* and 5 = *very often*) using three 10-item scales that was adapted from Stamm [21]. Participants rated their current work situation over the past month, with higher scores indicating greater levels of the respective construct. Sample items include: CS “*I feel invigorated after working with those I help*,” (α = 0.91, *M* = 35.18, *SD* = 6.77), burnout: “*I feel worn out because of my job as a nurse*,” (α = 0.84 *M* = 30.15, *SD* = 6.87), and STS: “*I feel depressed because of the traumatic experiences of the people I help*,” (α = 0.87, *M* = 27.42, *SD* = 7.56).

#### Personal network generator

Utilizing a similar method from Lee and colleagues [7], personal networks were elicited from respondents using an ego-centered cognitive social structure (ECSS) generator design [55]. ECSS is a methodology used to elicit information about how people mentally represent their personal social networks to examine how cognitive representation of personal support network influence perceived social support [55]. Given that people can bring to mind a different number of people, following prior research [7] a fixed-size requirement of four persons was implemented. This network size was chosen for several key reasons: (1) it provides sufficient statistical power for density calculations with 6 possible connections between alters, allowing meaningful distinction between sparse and dense networks [55]; and (2) it represents the optimal balance between network complexity and cognitive manageability for participants assessing relationships [55]. This fixed-size approach allowed us to control for any impact of the number of people on our outcome variables and to focus on associations of the structure (i.e., density) on our variable of interest (i.e., burnout). This also allowed us to capture a spontaneous network that is more chronically accessible in participants’ minds without having to sacrifice accuracy [55]. The social support prompt from the General Social Survey [56] was adapted to capture whom participants would go to for support:

From time to time, people receive social support from others. Looking back over the last six months -- who are the four people in your life that you go to for social support across different situations?

To better ascertain the nature of the relations between participants and their personal network members, participants were asked to indicate how much support they received from each alter (1 = *none at all* and 7 = *very much*) and how close they are to each alter (1 = *not at all close* and 7 = *very close*). Responses to these items across each personal network member were averaged to create indices of *average alter support* variable (α = 0.91, *M* = 5.47, *SD* = 1.52). Lastly, participants were asked to indicate their type of relationship (e.g., friend, family, romantic partner, coworker) with each alter.

#### Perceived network density

To measure perceived network density, participants were asked to report how close they perceived each pair of alters to be (whereby 1 = *not at all close* and 7 = *very close*). It is important to note that these judgements represented a bidirectional relation (i.e., undirected) between each pair of alters, meaning a single rating represented the relationship between two alters in both directions. Perceived network density was calculated and represented through two complimentary measures— weighted and unweighted network density— to capture both the strength and presence of connection for each respondent using the *average alter closeness* rating.

Weighted density accounts for the varying degrees of closeness between alters and was calculated as the overall sum of realized edge weights (connections between alters) divided by total possible numbers of edges:

$$\:\frac{\sum\:weights\:(i,\:j)}{\frac{1}{2}(N.\:\left(N-1\right)){\prime\:}}$$

where *i* and *j* are nodes within the network and *N* is the network size [55]. To provide a simpler binary measure of whether connections exist above a threshold, we calculate unweighted density by coding it as a dichotomous variable: edges with weights above the midpoint of the scale (>4) were coded as “1” to represent present, opposed to “0” to represent absent for edges whose weights were below the midpoint. For both measures, the focal participant (ego) was removed from density calculations to isolate the interconnected among alters. This means that in a network so that in a network where no alters know each other, the density would be 0, while a fully connected network where all alters are maximally close, the weighted density would approach 1. We used weighted density for our primary analyses as it provides more nuanced information about the strength of connections, while including unweighted density analyses as a robustness check (*M* = 0.72, *SD* = 0.31).

**Compassionate goals** were measured with 7 items from a preexisting scale by Crocker and Canevello [9], through a 5-point scale (1 = *Not at All*, 5 *= Very Much*). Participants were asked to read each statement in relation to their close relationships over the past month following established methodological conventions of previous research [9, 50, 51]. Items include, “In my relationship with others, I try to. be supportive of others,” and “. avoid being selfish or self-centered,” (α = 0.92, *M* = 4.04, *SD* = 0.85).

**Self-image goals** were measured with 6 items from Crocker and Canevello’s work [9], through a 5-point scale (1 = *Not at All*, 5 *= Very Much*). Items include, “In my relationships with others, I try to. avoid the possibility of being wrong,” and “. get others to recognize or acknowledge your positive qualities,” (α = 0.79, *M* = 3.19, *SD* = 0.86).

### Data analysis

All analyses were performed using SPSS version 26.0. Consistent with prior work [7], we excluded 28 responses who listed fewer than four alters or who failed to complete the perceived network density measure. The final sample size used for subsequent analyses was 220. First, regression analysis was conducted to test the relationship between compassionate goals and perceived personal network density (H1), and burnout levels (H2). As perceived personal network density from alter was proposed to mediate the association between compassionate goals and burnout levels (H3), we utilized Model 4 of Hayes’ Macro Process 4.1 to test mediation [57]. The model tested how compassionate goals were correlated with burnout, and 95% confidence intervals (CIs) were reported to indicate the significance of the mediation (i.e., indirect association). If zero is not included in the 95% CI, the indirect association is significant.

Similarly, to investigate the associations of self-image goals, we conducted another set of regression analyses to test the relationship between self-image goals and perceived personal network density (H4), as well as burnout levels (H5). Mediation was tested by using Model 4 from Hayes’ Macro Process 4.1 [57] (H6). As before, 95% CI was reported to indicate significance of the indirect association.

## Results

Prior to hypothesis testing, we examined our data for multicollinearity and normality. Variance Inflation Factor (VIF) values for all variables were below 2, indicating no concerning levels of multicollinearity. All variables demonstrated acceptable levels of normality based on skewness and kurtosis values (all within ± 2). Table 1 presents the correlation of all variables measured in this study. Because age and gender were significantly correlated with our key variables, we decided to control for both in all our analyses.

**Table 1:** Zero-order bivariate correlations among key variables

	Variables	M	SD	1	2	3	4	5	6	7	8	9	10	
1.	Compassionate goals	4.04	0.85	--										
2.	Self-image goals	3.19	0.86	− 0.23**	--									
3.	Weighted network density	0.72	0.31	0.75**	− 0.42**	--								
4.	Unweighted network density	0.79	0.40	0.70**	− 0.27**	0.76**	--							
5.	Compassion Satisfaction	35.18	6.77	0.37**	− 0.111	0.35**	0.27**	--						
6.	Burnout	30.15	6.87	− 0.62**	0.47**	− 0.68**	− 0.52**	− 0.55**	--					
7.	Secondary Traumatic Stress	27.42	7.56	− 0.23**	0.29**	− 0.36**	− 0.29**	− 0.44**	0.53**	--				
8.	Average alter support	5.47	1.52	0.69**	− 0.30**	0.65**	0.61**	0.33**	− 0.63**	− 0.31**	--			
9.	Age	33.13	12.03	0.12	− 0.21**	0.15	0.09	0.31**	− 0.27**	− 0.32**	0.15	--		
10.	Gender^a^	1.81	0.48	0.20**	0.06	0.08	0.14	0.06	− 0.06	− 0.17*	0.15	0.01	--	

Note: ^a^0 = Male, 1 = Female; *p < .01; ** p < .001 (two-tail tests)

### Investigating compassionate goals

We conducted a set of multiple regression analyses to investigate the hypotheses that compassionate goals would be positively correlated with perceived personal network density and lower levels of reported burnout levels (i.e. H1 and H2). Results indicated that compassionate goals significantly correlated with perceived personal network density, *β* = 0.50, *p* < .001, even after controlling for perceived alter support (*β* = 0.18, *p* = .04), age (*β* = 0.06, *p* = .46), gender (*β* = − 0.04, *p* = .52), income (*β* = − 0.01, *p* = .82), and country (*β* = − 0.03, *p* = .71). Additionally, compassionate goals showed a significant direct negative association on burnout, *β* = -1.96, *p* = .04, 95% CI = [-3.48, − 0.08], suggesting that higher compassionate goals had a negative correlation with lower burnout levels. Perceived personal network density was negatively correlated with reported burnout, *β* = -1.96, SE = 0.71, 95% CI = [-3.48, − 0.08], *β* = − 0.18, *p* = .04. These results support H1 and H2. Table 2 presents the regression coefficients when the variable, *compassionate goals*, is entered as a variable for perceived personal network density and burnout.^1^

**Table 2:** Regression coefficients influencing perceived personal network density and burnout from compassionate goals

	Perceived Network Density					Burnout				
	B	*SE*(B)	*β*	*p*	CI	B	*SE*(B)	*β*	*p*	CI
Gender^a^	− 0.03	0.04	− 0.04	0.52	− 0.11, 0.05	1.36	0.91	0.08	0.26	-0.44, 3.16
Age	0.01	0.00	0.06	0.46	− 0.01, 0.01	− 0.12	0.04	− 0.18*	0.01	− 0.20, − 0.02
Income	0.02	0.01	− 0.01	0.82	− 0.01, 0.02	− 0.02	0.19	− 0.01	0.92	− 0.39, 0.35
Country^b^	0.01	0.00	− 0.03	0.71	− 0.00, 0.01	− 0.04	0.03	− 0.11	0.22	− 0.10, 0.02
Perceived alter support	0.06	0.02	0.18*	0.04	0.00, 0.10	− 0.27	0.10	− 0.40**	< 0.001	-0.47, -0.07
Compassionate goals	0.22	0.03	0.50**	< 0.001	0.16, 0.28	-2.08	0.71	-1.96*	0.04	-3.48, − 0.08
*F*(*df*), *AdjR*^*2*^	15.32** (6,213), 0.38					14.99**(6,213), 0.41				

Note: ^a^0 = Male, 1 = Female; ^b^0= United States, 1 = All others, *p < .01, **p < .001

To test H3 (i.e. compassionate goals would be positively correlated with personal network density, which in turn, would have a negative correlation with burnout levels), we conducted a mediation analysis using Model 4 of the PROCESS macro from SPSS [57] with 10,000 corrected bootstrap samples. Consistent with our hypothesis, the results revealed a significant indirect association between compassionate goals on burnout through personal network density, effect = -1.00, 95% CI = [-1.85, -0.35].

### Investigating self-image goals

To test H4 and H5, we conducted additional set of regression analyses. Results indicated that self-image goals were negatively correlated with perceived personal network density, *β* = − 0.18, *p* = .04, while controlling for perceived alter support (*β* = 0.39, *p* < .001), age (*β* = 0.00, *p =* .98), gender (*β* = − 0.04, *p* = .64), income (*β* = 0.03, *p* = .70), and country (*β* = 0.01, *p* = .96). H4 was supported. Next, perceived personal network density was negatively correlated with levels of burnout, *β* = -9.01, SE = 2.2, 95% CI [-13.36, -4.65], *β* = − 0.32, *p* < .001. Additionally, self-image goals demonstrated a significant direct positive association on burnout, *β* = 0.24, *p* < .001, 95% CI = [0.84, 3.80], indicating that higher self-image goals were positively correlated with burnout levels. H5 was supported. Table 3 presents the regression coefficients when *self-image goals* is entered as a variable for perceived personal network density and burnout.

**Table 3:** Regression coefficients influencing perceived personal network density and burnout from self-image goals

	Perceived Network Density					Burnout				
	B	*SE*(B)	*β*	*p*	CI	B	*SE*(B)	*β*	*p*	CI
Gender^a^	− 0.03	0.05	− 0.04	0.64	− 0.13, 0.07	1.36	1.33	0.07	0.31	-1.3, 3.99
Age	0.00	0.01	0.00	0.98	− 0.00, 0.01	− 0.09	0.05	− 0.15	0.06	− 0.20, 0.00
Income	0.00	0.01	0.03	0.70	− 0.01, 0.02	− 0.12	0.22	− 0.04	0.58	− 0.55, 0.31
Country^b^	0.00	0.00	0.02	0.96	− 0.00, 0.01	− 0.04	0.03	− 0.09	0.25	− 0.01, 0.03
Perceived alter support	0.12	0.02	0.39**	< 0.001	0.08, 0.16	-3.31	0.52	− 0.47**	< 0.001	-4.36, -2.27
Self-image goals	− 0.06	0.03	− 0.18*	0.04	− 0.12, − 0.00	2.32	0.75	0.24*	0.00	0.84, 3.80
*F*(*df*), *AdjR*^*2*^	6.65** (6,213), 0.22					13.49**(6,213), 0.39				

Note: ^a^0 = Male, 1 = Female; ^b^0= United States, 1 = All others, *p < .05, **p < .001

To test H6 (i.e. self-image goals would be negatively correlated with personal network density, which in turn, would be positively correlated with higher burnout levels), we conducted a mediation analysis using Model 4 of the PROCESS macro from SPSS [57] with 10,000 corrected bootstrap samples. The results revealed a significant indirect association between self-image goals and burnout through perceived personal network density, effect = 0.36, 95% CI = [0.12, 0.72].

## Discussion

A growing body of research indicates that the ways in which people perceive their social network has implications for social resources [7, 41, 42]. Building on this work, the current study examined how two distinct interpersonal goals may relate to the perception of the density of one’s personal network of nurses, as well as its implications for burnout. Consistent with our hypotheses, results indicated that interpersonal goals correlated differentially with perceived personal network density and burnout levels. Specifically, compassionate goals were significantly correlated with perceiving a more interconnected personal network, and both were negatively correlated with burnout levels. Conversely, self-image goals were positively correlated with burnout, and perceived network sparsity statistically mediated this association.

The current research makes several contributions. First, it extends prior research on cognitive social structure [40, 43] by examining how social motivation (i.e., interpersonal goals) may be correlated with perceiving on one’s personal network as dense (vs. sparse). Results from this study suggest that people who were more likely to promote the well-being of others may view their personal network as interconnected, while the opposite was found for those whose primary focus is enhancing others’ regards of them.

Second, the current research contributes to research on the mental well-being of healthcare workers [39] by testing how interpersonal goals and personal network perception research may shed light on ways to maximize one’s existing social resources and potentially mitigate burnout. Broadly, the ability for a person to leverage their close relationships for social resources is important. As mentioned, people with compassionate goals are more likely to reap long-term benefits from their relationships, while the opposite occurs for those with self-image goals [9, 11, 58]. Interestingly, these findings did not change even after accounting for the quality of support received from personal network, suggesting that interpersonal goals may be linked to burnout levels (via perceived network density) beyond support quality.

Third, the present study contributes to research on interpersonal goals in multiple ways. To date, most work on interpersonal goals have been limited to studying convenience samples (e.g., college students). While some initial work has explored these goals in nursing contexts (e.g., Redfield et al., 2016 [59] examined interpersonal goals among nursing students in relation to attitudes toward older adults), our study extends this work by examining practicing nurses in high-stress environments. By focusing on nurses, we were able to generalize the interpersonal motivation goals framework to a unique context—one that involves working in a high-stress environment that demands empathy and concern towards others’ physical and mental well-being. Furthermore, most post-pandemic work has focused on identifying what individual traits are associated with lower levels of burnout among nurses [39]. By highlighting the role of interpersonal goals, this study shifts the focus towards motivational mechanisms associated with burnout. Additionally, while previous studies that used interpersonal goals framework focused on dyadic level interactions, this study is the first to extend its framework to a network-level. To that end, future research should consider how the structure of our personal network, when evoked, shapes our behaviors.

By focusing on the cognitive structure of one’s network, this study provides preliminary evidence regarding how thinking about one’s personal network as dense (vs. sparse) could be associated with well-being. In this vein, future studies should consider how findings from this current work may inform a cost-effective intervention strategy that encourages nurses to think about their personal network as more dense or nudges them to have more compassionate goals [60]. Additionally, future research should consider how the activation of these networks could optimize prosocial behavior such as the provision of support to others. That is, do people who perceive a denser (vs. sparse) personal network readily provide support to others in times of crisis? Conversely, would thinking of a sparse personal network lower the likelihood of providing resources to others?

There were some limitations of this study. First, because the data are cross-sectional, we cannot infer causal or temporal effects among interpersonal goals, perceived network density, and burnout levels. For instance, it is possible that demands from the job may influence nurses to adopt compassionate goals to acquire support from their personal network, or that feelings of mental exhaustion may relate to perceiving a more connected personal network for support. Future studies are needed to enhance the understanding of these complex relations via experimental manipulations or through a longitudinal study. Second, a fixed name generator methodology was used to assess participants’ personal network. While implementing a four-alter set of nodes helped us to control for size-based confounds, we acknowledge that this may not accurately represent a structure that would otherwise be produced without any limit. Nonetheless, previous work has suggested that a fixed-choice generator produces a network fairly similar to one created by a free-choice generator as those alters that come to mind are most salient to the ego [42, 55, 61]. Third, although we oversampled to ensure sufficient power, future studies should attempt to recruit more nurses and replicate our findings. Related, we acknowledge that the utilization of social media for recruitment might have excluded nurses who do not use social media. It is possible that systematic differences such as socioeconomic status and personal characteristics [62] exist between nurses who use social media versus non-users—factors that could have bias the current sample, limiting the generalizability of our results. Future studies should therefore consider other recruitment techniques to address this limitation. Finally, although our study controlled for country and found no significant associations with key outcomes, this limitation underscores the need for more nuanced cross-cultural research. While our findings offer valuable insights into compassionate goals and burnout, future studies could illuminate the complex ways cultural context might shape these psychological processes among healthcare professionals.

## Conclusion

The present research contributes to the ongoing discussion on factors that influence burnout amongst nurses through the integration of two disparate bodies of literature (i.e., interpersonal goals and network perception). Interpersonal goals were associated with how people perceived the interconnectedness among their core personal network members, which were associated with burnout levels. These findings point to the potential importance of interpersonal goals and network perceptions as areas for future research on strategies to support well-being in high-stress settings such as healthcare.

## Data Availability

Restrictions apply to the availability of these data due to privacy concerns. However, the dataset for the current study is available from the corresponding author on reasonable request.

## Footnotes

1 Given that compassionate and self-image goals were significantly correlated (*r* = − .23, *p* < .001), we centered the goals and entered them simultaneously as independent variables in our analyses, controlling for each other. When both goals were included, the effect of compassionate goals on perceived personal network density remained significant (β = 0.43, *p* < .001, 95% CI = [0.15, 0.27]), with a slight attenuation from the original model. Self-image goals also demonstrated a significant negative effect on perceived network density (β = − 0.19, *p* = .03, 95% CI = [-0.11, − 0.01]). These results suggest the robust relationship between both goal types and perceived personal network density, even when controlling for each other.

## Abbreviations

CF: Compassion Fatigue
CI: Confidence Interval
COVID: Coronavirus Disease 2019
CS: Compassion Satisfaction
CSS: Cognitive Social Structure
ECSS: Ego-Centered Cognitive Social Structure
ID: Identification number
IRB: Institutional Review Board
PROCESS: Hayes’ PROCESS macro for mediation analysis
ProQoL: Professional Quality of Life scale
SD: Standard Deviation
SE: Standard Error
SPSS: Statistical Package for the Social Sciences
STS: Secondary Traumatic Stress
VIF: Variance Inflation Factor (VIF)
